# N-terminal pro-brain natriuretic peptide and cardiovascular or all-cause mortality in the general population: A meta-analysis

**DOI:** 10.1038/srep41504

**Published:** 2017-01-30

**Authors:** Zhaohua Geng, Lan Huang, Mingbao Song, Yaoming Song

**Affiliations:** 1 Department of Cardiology, The Second Affiliated Hospital, Third Military Medical University, Chongqing 400037, China

## Abstract

The prognostic role of N-terminal pro-brain natriuretic peptide (NT-proBNP) in the general population remains controversial. We conducted this meta-analysis to investigate the association between baseline NT-proBNP concentrations and cardiovascular or all-cause mortality in the general population. PubMed and Embase databases were systematically searched from their inception to August 2016. Prospective observational studies that investigated the association between baseline NT-proBNP concentrations and cardiovascular or all-cause mortality in the general population were eligible. A summary of the hazard ratio (HR) and 95% confidence interval (CI) of mortality were calculated by the highest versus the lowest category of NT-proBNP concentrations. Eleven studies with a total of 25,715 individuals were included. Compared individuals in the highest with those in the lowest category of NT-proBNP, the pooled HR was 2.44 (95% CI 2.11–2.83) for all-cause mortality, 3.77 (95% CI 2.85–5.00) for cardiovascular mortality, and 2.35 (95% CI 1.45–3.82) for coronary heart disease mortality, respectively. Subgroup analyses indicated that the effects of NT-proBNP on the risk of cardiovascular mortality (RR 2.27) and all-cause mortality (RR 3.00) appeared to be slightly lower among men. Elevated NT-proBNP concentrations appeared to be independently associated with increased risk of cardiovascular and all-cause mortality in the general population.

N-terminal pro-brain natriuretic peptide (NT-proBNP) is a prohormone with a 76 amino acid N-terminal inactive protein that is cleaved from the molecule to release brain natriuretic peptide (BNP)^1^. BNP and NT-proBNP are synthesized in response to ventricular stretch and ischemic injury^2^. Measurement of circulating BNP and NT-proBNP concentrations have been recommended in the diagnosis and management of heart failure^3^,^4^. Determining the NT-proBNP concentrations is recommended because of its more stable form and longer half-life^5^. Even in the absence of heart failure, elevated circulating NT-proBNP concentrations have also emerged as a serologic marker for the assessment of cardiovascular disease^6^.

Numerous studies have assessed the predictive value of circulating NT-proBNP concentrations in the general population^7^,^8^,^9^,^10^,^11^,^12^,^13^,^14^,^15^. However, the role of NT-proBNP as a predictor of mortality in the general population is conflicting^16^,^17^. In addition, the magnitude of the association between elevated NT-proBNP concentrations and risk of mortality varied across studies due to distinct study designs and studied populations. Currently, no previous a meta-analysis has evaluated this association in the general population. We therefore performed the current meta-analysis of the available prospective observational studies to investigate the association between baseline NT-proBNP concentrations and cardiovascular or all-cause mortality in the general population.

## Results

### Literature search and study characteristics

**Figure 1:**
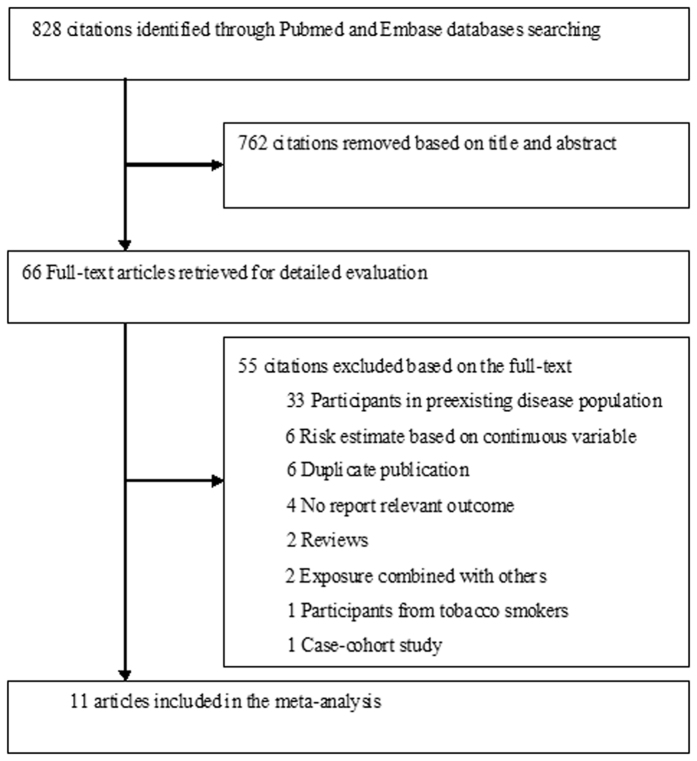
Flow chart of the study selection process.

The initial electronic search yielded 828 citations. After screening the titles and abstracts, 66 articles were reviewed for more detailed evaluation, and 55 articles were further excluded mainly due to participants from a high cardiovascular risk or preexisting disease population. Finally, 11 studies^7^,^8^,^9^,^10^,^11^,^12^,^13^,^14^,^15^,^16^,^17^ were selected in this meta-analysis. The flow chart of the study selection is shown in Fig. 1.

The main characteristics of the included studies are summarized in Table 1. The included studies were published from 2005 to 2016. Of 11 studies, six studies^7^,^8^,^9^,^12^,^14^,^16^ were conducted in Europe, three^10^,^13^,^17^ in the USA, and two in Asia^11^,^15^. Sample sizes ranged from 506 to 11,193 with a total of 25,715 participants. Follow-up durations varied from 4.8 to 11.9 years. Three studies^7^,^8^,^11^ only consisted of men. All the included studies measured NT-proBNP concentrations by an electrochemiluminescence immunoassay performed on a Roche analyzer. The overall quality of most studies was high with NOS stars ranging from 5 to 8.

### All-cause mortality

**Figure 2:**
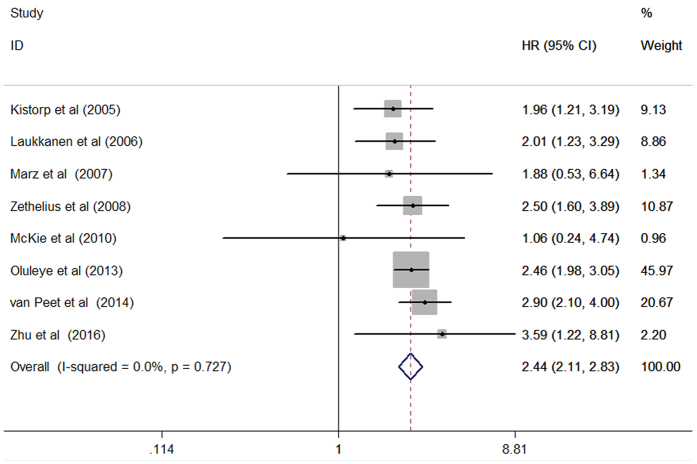
Forest plots showing pooled hazard ratio and 95% confidence interval of all-cause mortality comparing the highest with the lowest concentrations of N-terminal pro-brain natriuretic peptide in a fixed-effect model.

Data on all-cause mortality were available from 8 studies^7^,^8^,^9^,^13^,^14^,^15^,^16^,^17^. A total of 2,623 total death events were reported from 16,653 participants. As shown in Fig. 2, there was no evidence of significant heterogeneity across studies (I^2^ = 0%, p = 0.727). When compared with the lowest NT-proBNP concentrations, individuals with the highest NT-proBNP concentrations at baseline were significantly associated with an increased risk of all-cause mortality (HR 2.44; 95% CI 2.11–2.83) in a fixed-effect model. Evaluation of publication bias indicated that the both of Begg’s test (p = 0.266) and Egger’s test (p = 0.330) were not significant for the all-cause mortality. Sensitivity analyses showed that there were few changes in pooled risk estimates when any single study was removed at each turn.

### Cardiovascular mortality

**Figure 3:**
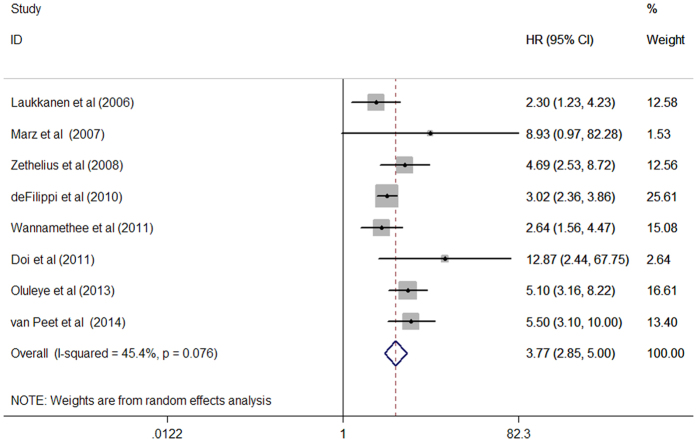
Forest plots showing pooled hazard ratio and 95% confidence interval of cardiovascular mortality comparing the highest with the lowest concentrations of N-terminal pro-brain natriuretic peptide in a random effect model.

Eight studies^8^,^9^,^10^,^11^,^12^,^13^,^14^,^16^ provided the data on cardiovascular mortality. A total of 1,396 cardiovascular death events were reported from 22,887 participants. As shown in Fig. 3, a significant degree of study heterogeneity was noted (I^2^ = 45.4%, p = 0.076). When compared with the lowest NT-proBNP concentrations, individuals with the highest concentrations of NT-proBNP were associated with greater risk of cardiovascular mortality (HR 3.77; 95% CI 2.85–5.00) in a random-effects model. No evidence of publication bias was observed (p = 0.902 for Begg’s tests; p = 0.141 for Egger’s test).

### Coronary heart disease (CHD) mortality

**Figure 4:**
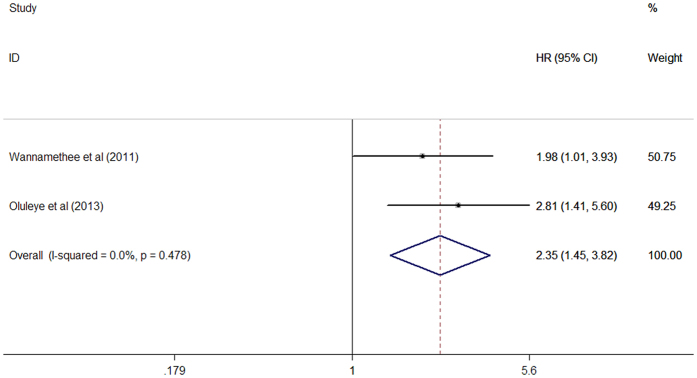
Forest plots showing pooled hazard ratio and 95% confidence interval of coronary heart disease mortality comparing the highest with the lowest concentrations of N-terminal pro-brain natriuretic peptide in a fixed-effect model.

Data on CHD mortality were only available from 2 studies^12^,^13^. As shown in Fig. 4, there was no evidence of significant heterogeneity between two studies (I^2^ = 0%, p = 0.478). The pooled HR for mortality due to CHD was 2.35 (95% CI 1.45–3.82) in a fixed-effect model.

### Subgroup analyses

Subgroup analyses indicated that the association between elevated NT-proBNP concentrations and risk of cardiovascular and all-cause mortality was consistently observed in each subgroup (Table 2). The risk of cardiovascular and all-cause mortality was higher in studies with a follow-up duration ≤5 years or mean age ≥70 years. The risk of cardiovascular and all-cause mortality was lower in studies enrolling only men or use of NT-proBNP cutoff value.

## Discussion

NT-proBNP has been shown to predict poor prognosis in a variety of settings, including heart failure^18^, acute coronary syndromes^19^, stable coronary artery disease^20^, or stroke^21^. This meta-analysis goes beyond these established cardiovascular diseases and especially extends to the setting of the general population. The current meta-analysis demonstrates that elevated NT-proBNP concentrations appeared to be independently associated with increased risk for CHD, cardiovascular and all-cause mortality in the general population. Individuals in the highest NT-proBNP concentrations significantly increased 2.35-fold CHD mortality, 3.77-fold cardiovascular mortality, and 2.44-fold all-cause mortality after adjustment for other traditional risk factors.

Age, gender, renal impairment, and obesity may affect the circulating concentrations of NT-proBNP. NT-proBNP concentrations varied by the age of the study population^11^,^22^. This meta-analysis included studies spanning a wide range of age. Our subgroup analysis showed that the prognostic value of cardiovascular and all-cause mortality risk was stronger for participants with mean age ≥70 years than those with a mean age <70 years, particularly for cardiovascular mortality (RR 5.10 vs.3.40). Zhu *et al*.’s study^15^ also suggested that NT-proBNP concentrations were an independent predictor of all-cause mortality in participants with age >65 years but not age <65 years. Stratified analysis by gender showed that the effects of elevated NT-proBNP concentrations on cardiovascular and all-cause mortality risk appeared to be slightly lower in men than the both gender groups. This result may be explained by the women had significantly higher concentrations of NT-proBNP than the men^23^. Therefore, gender specific analysis of NT-proBNP concentrations on subsequent mortality risk is required in the future studies. In addition, the risk of cardiovascular and all-cause mortality was higher in studies with a follow-up duration ≤5 years than in those with >5 years of follow-up, suggesting death events mainly occured in the early follow-up duration.

NT-proBNP was at least partially cleared from the circulation by the kidney^24^. Circulating concentrations of NT-proBNP are typically higher in patients with chronic kidney disease (CKD) than in those without CKD^25^,^26^. Therefore, CKD may be an important confounding factor that affecting the association between NT-proBNP and mortality risk. However, our subgroup analysis revealed that whether adjustment for renal function was not found to significantly alter the prognostic value of the NT-proBNP. These findings suggested the association between NT-proBNP and mortality risk was independent of CKD.

Obesity must be taken into account for clinical interpretation of NT-proBNP. There was a paradoxical association between obesity and prognosis in patients with heart failure^27^. Overweight and obese adults had a lower NT-proBNP concentrations than those in the normal weight^28^,^29^. NT-proBNP concentrations appeared to be inversely correlated with obesity^30^. The inverse relationship between the NT-proBNP concentrations and body mass index (BMI) might be explained by an increase in the degradation of the adipose tissue peptide^31^. Therefore, obesity may have confounded the association of NT-proBNP concentrations with cardiovascular or all-cause mortality. However, we could not conduct a subgroup analysis by obesity (e.g. BMI ≥ 30 kg/m^2^ vs. BMI < 30 kg/m^2^) because the included studies did not report the risk estimate by the category of body weight. Future studies are recommended to report risk estimate of cardiovascular or all-cause mortality with the category of BMI or waist circumference.

Several studies also investigated the relationship between circulating NT-proBNP concentrations and mortality risk based on continuous data analysis. Per 1 SD or per 1 unit increase in log NT-proBNP concentrations was associated with an increase risk of cardiovascular and all-cause mortality after adjustment for other traditional risk factors^32^,^33^,^34^,^35^,^36^. In addition, individuals with increasing NT-proBNP concentrations (≥100%) also had markedly increased all-cause mortality compared with those with unchanged^37^,^38^. Findings in continuous NT-proBNP analysis further supported the prognostic value of NT-proBNP on the mortality risk.

Data on comparison of NT-proBNP relative to BNP concentrations as predictors of mortality in the general population were unavailable. NT-proBNP seemed to be superior to BNP for predicting cardiovascular events in the general population^39^ and patients with stable coronary heart disease^40^. This finding may be correlated to NT-proBNP has a longer half life than BNP and higher plasma concentrations^41^. Several possible mechanisms can explain the prognostic value of circulating NT-proBNP concentrations in the general population. First, higher NT-proBNP concentrations may reflect the presence of structural heart disease or cardiac remodeling resulting from increased cardiac stretch^42^. Second, elevated NT-proBNP concentrations may link with the degree of systemic atherosclerosis^43^.

Several potential limitations should be mentioned. First, circulating concentrations of NT-proBNP were determined at a single measurement at baseline and without observed the dynamic changes. The concentrations NT-proBNP could be changed by modifications in lifestyle or medication during the follow-up^10^ and misclassification of NT-proBNP category was possible. Therefore, NT-proBNP might not optimally stratify long-term clinical endpoints. Second, the findings from the subgroup analysis may be reliable because of the relatively small number of included studies analyzed. Third, the majority of the included studies pertained to the older participants; hence generalization of our findings to the middle-aged individuals might be limited. Fourth, results of publication bias may be unreliable mainly due to the studies’ number of cardiovascular and all-cause mortality was less than 10. Finally, as for the thresholds for higher concentrations of NT-proBNP markedly varied across studies, we were unable to define the optimal thresholds for NT-proBNP.

In conclusion, this meta-analysis suggests that individuals with elevated NT-proBNP concentrations appeared to be independently associated with an increased risk for CHD, cardiovascular and all-cause mortality in the general population. Future more well-designed prospective studies are necessary to investigate the gender-specific effects of NT-proBNP on mortality risk.

## Methods

### Search strategy

This meta-analysis was performed according to the guidelines of the Meta-analysis of Observational Studies in Epidemiology^44^. A systematic electronic literature search was conducted in PubMed and Embase databases from inception to August 2016 without restriction. The following search terms were used: N-terminal pro-brain natriuretic peptide OR NT-proBNP OR BNP AND “mortality” OR “death” AND “prospective studies” OR “longitudinal study” OR “follow-up”. Reference lists from identified studies were manually scanning to identify any additional articles.

### Study selection

The eligible studies had to satisfy the following inclusion criteria:1) prospective observational design with participants in the general population (participants not from disease-specific populations); and 2) reported adjusted hazard ratio (HR) or risk ratio (RR) and corresponding 95% confidence interval (CI) of cardiovascular or all-cause mortality comparing the highest to the lowest category of baseline NT-proBNP concentrations. If multiple articles from the same population, the study with larger sample size and longer follow-up duration were selected. Exclusion criteria included: 1) participants from a high cardiovascular risk or preexisting disease population; 2) circulating BNP concentration as exposure; 3) only reported risk estimate based on continuous variable of NT-proBNP concentrations; and 4) conference abstracts, review, or case-control study.

### Data extraction and quality assessment

Data extracted from each study included: first author’s surname, publication year, geographic region, study design, sample sizes, mean age or age range of the participants, gender, method of NT-proBNP detection, cutoff value of NT-proBNP, number of death events, fully adjusted HR with corresponding 95% CI, duration of follow-up, and degree of adjustment for potential confounders. Two independent authors (ZH Geng and L Huang) independently extracted the data. Disagreements were resolved by discussion or consensus from a third author (MB Song). If the original data were incomplete, the corresponding author was contacted by e-mail. Methodological quality was evaluated with the 9-star Newcastle-Ottawa Scale (NOS) for the cohort studies^45^. Domains of quality assessment included selection of the study groups, comparability of groups, and ascertainment of outcomes. If the studies achieved six stars or more, we grouped them as high quality.

### Statistical analyses

The most fully adjusted HR or RR and 95% CI were used to calculate the pooled effects. HR and RR were assumed to approximate the same measure of the risk estimate. The pooled risk estimate was calculated by the highest versus the lowest category of NT-proBNP concentrations. Cochrane Q test and I^2^ statistics were used to measure the heterogeneity across studies. Statistically significant heterogeneity was defined as p < 0.10 for the Cochrane Q test and I^2^ > 50%. A random effect model was used when there was evidence of significant heterogeneity; otherwise, a fixed-effects model was utilized^46^. Subgroup analyses were conducted by the number of participants, region, duration of follow-up, and NOS scores. We conducted a sensitivity analysis to assess the robustness of the overall risk estimate by sequentially excluding a single study at each turn. Potential publication bias was evaluated using the Begg’s rank correlation test^47^ and Egger’s regression test^48^, with both *P* values > 0.10 considered as not significant. All analyses were performed with Stata software 12.0 (Stata, College Station, TX, USA).

## Additional Information

**How to cite this article**: Geng, Z. *et al*. N-terminal pro-brain natriuretic peptide and cardiovascular or all-cause mortality in the general population: A meta-analysis. *Sci. Rep.* **7**, 41504; doi: 10.1038/srep41504 (2017).

**Publisher's note:** Springer Nature remains neutral with regard to jurisdictional claims in published maps and institutional affiliations.

## Figures and Tables

**Table 1:** Baseline characteristics of the included studies.

Author/year	Region	Study name	Design	Sample sizes (% male)	Age (years)	NT-proBNP comparison	Follow-up (years)	No. death/HR (95% CI)	Adjustment for variables	Overall NOS
Kistorp *et al*.^7^	Denmark	—	Population-based prospective study	626 (42.3)	67.9 ± 10.6	>80th percentile vs. others; >655.4 pg/ml vs. ≤655.4 pg/ml	5	Total death:94 1.96 (1.21–3.19)	Age, sex, current smoking, DB, hypertension and ischemic heart disease, TC, and creatinine	8
Laukkanen *et al*.^8^	Finland	KIHD	Prospective study	905 (100)	55.8 ± 6.6	>90th percentile vs. others; >133.4 pmol/L vs. ≤133.4 pmol/L	9.8	CV death:58; 2.3 (1.23–4.23); Total death:110; 2.01 (1.23–3.29)	Age, smoking, DB, SBP, family history of CHD, presence or absence of CHD, BMI, LDL, HDL, CRP, creatinine, and antihypertensive drugs.	7
März *et al*.^16^	Germany	LURIC	Prospective study	506 (NP)^	61.1 ± 10.8	Tertile 3 vs. tertile 1; ≥400 ng/L vs. <100 ng/L	5.45	CV death:16 8.93 (0.97–82.28); Total death:32; 1.88 (0.53–6.64)	Age, sex, DB, CRP, BMI, smoking, hypertension, dyslipidemia, eGFR, presence or absence of CAD on angiography, previous MI, use of beta-blockers, ACEIs, ARBs, CCBs, diuretics, antiplatelet drugs, lipid-lowering agents, revascularization at baseline, and LV function	6
Zethelius *et al*.^9^	Sweden	ULSAM	Prospective community-based study	661 (100)#	71 ± 0.6	Cutoff value; >309 ng/liter vs. ≤309 ng/liter	10.0	CV death:54 4.69 (2.53–8.72); Total death:149; 2.50 (1.60–3.89)	Age, SBP, use or non use of antihypertensive or lipid- lowering agent, TC, HDL, DB, smoking, and BMI	8
deFilippi *et al*.^10^	USA	CHS	Prospective community-based study	2,975 (40.6)	72.7 ± 5.5	Quintile 5 vs. quintile 1; >267.7 pg/ml vs. <47.5 pg/ml	11.9	CV death:539 3.02 (2.36–3.86)	Age, sex, race, smoking, TC, HDL, SBP, hypertension., DB, BMI, CHD, renal function, any major ECG abnormality, use of ACEIs/ARBs, beta-blockers, and diuretics	7
McKie *et al*.^17^	USA	REP	Prospective community-based cohort	703 (47)*	56 ± 7	>80th percentile vs. others; >196 pg/ml for women and >125 pg/ml for men.	10	Total death:19 1.06 (0.24–4.74)	Age, sex, and BMI.	5
Doi *et al*.^11^	Japan	Hisayama	Population-based prospective study	3,104 (42.0)	61.3 ± 12.4	Quintile 4 vs. quintile 1; ≥400 pg/ml vs. <55 pg/ml	5	CV death:48 12.87 (2.44–67.75)	Age, sex, SBP, electrocardiogram abnormalities, eGFR., BMI, DB, TC, HDL, smoking, alcohol, and regular exercise	7
Wannamethee *et al*.^12^	UK	BRHS	Prospective study	2,983 (100)	60–79	Quintile 4 vs. quintile 1; ≥151 pg/ml vs. ≤40 pg/ml	9	CV death:223 2.64 (1.56–4.47); CHD death:119 1.98 (1.01–3.93)	Age, smoking, physical activity, alcohol intake, BMI, SBP, HD, TC, FEV1, DB, CRP, anemia, atrial fibrillation, and eGFR	8
Oluleye *et al*.^13^	USA	ARIC	Prospective cohort study	11,193 (NP)	45–64	Quintile 5 vs. quintile 1; ≥159 pg/ml vs. ≤27.4 pg/ml	9.9	CV death:358 5.10 (3.16–8.22); CHD death:138 2.81 (1.41–5.60); Total death:1,909 2.46 (1.98–3.05);	Age, gender, race, BMI, TC, HDL, diet, sport index, smoking, drinking, hormone use, SBP, antihypertensive medication, DB, FEV1, eGFR., Hs-CRP, and troponin T. (total mortality was adjusted for history of cancer, CVD, stroke, HF, and respiratory disease.	8
van Peet *et al*.^14^	The Netherlands	Leiden 85-plus	Prospective cohort study	560 (34)	≥ 85	Tertile 3 vs. tertile 1; >649 pg/ml vs. <201 pg/ml in men and >519 pg/ml vs. <204 pg/ml in women	5	CV death:100 5.5 (3.1–10); Total death:258 2.9 (2.1–4.0)	Age, sex, microalbuminuria, eGFR, prevalent CVD, DB, SBP, use of antihypertensive drugs, smoking, BMI, TC, HDL, and lipid medication use.	6
Zhu *et al*.^15^	China	—	Community-based prospective survey	1,499 (42)	61.4 ± 11.4	Quintile 4 vs. quintile 1; ≥81.9 pg/ml vs. <19.8 pg/ml	4.8	Total death:52 3.59 (1.22–8.81)	Age, sex, current smoking, BMI, SBP, DBP, FBG, TC, HDL-C, LDL-C, eGFR, high-sensitivity CRP, and homocysteine.	6

Abbreviations: BMI, body mass index; HR, hazard ratio; CI, confidence interval; NP, not provided; SBP, systolic blood pressure; DBP, diastolic blood pressure; DB, diabetes mellitus; TG, triglyceride; LDL, low-density lipoprotein; HDL, high-density lipoprotein; TC, total cholesterol; CV, cardiovascular; CVD, cardiovascular disease; CHD, coronary heart disease; MI, myocardial infarction; eGFR, estimated glomerular filtration rate; ACR, albumin to creatinine ratio; ACEI, angiotensin converting enzyme inhibitors; CCBs, calcium channel blockers; ARBs, angiotensin receptor blockers; NOS, Newcastle-Ottawa Scale; NT-proBNP, N-terminal prohormone B-type natriuretic peptide; CRP, C-reactive protein; FEV1, forced expiratory volume in 1 second; KIHD, Kuopio Ischemic Heart Disease Risk Factor Study; ARIC, Atherosclerosis Risk in Communities; CHS, Cardiovascular Health Study; BRHS, British Regional Heart Study; REP, Rochester Epidemiology Project; LURIC. Ludwigshafen Risk and Cardiovascular Health Study; ULSAM, Uppsala Longitudinal Study of Adult Men.

^#^healthy normal individuals; ^No angiographic CAD.

**Table 2:** Subgroup analyses on cardiovascular and all-cause mortality.

Subgroup	No. of studies	Pooled HR	95% CI	Heterogeneity between studies
1. All-cause mortality				
Sample size				
≥1,000	2	2.50	2.03–3.09	p = 0.575; I^2^ = 0%;
<1,000	6	2.39	1.95–2.93	p = 0.464; I^2^ = 0%
Mean age				
≥70 years	2	2.76	2.12–3.58	p = 0.596; I^2^ = 0%
<70 years	6	2.31	1.94–2.76	p = 0.702; I^2^ = 0%
Follow-up duration				
>5 years	5	2.36	1.98–2.82	p = 0.764; I^2^ = 0%
≤5 years	3	2.63	2.03–3.41	p = 0.342; I^2^ = 6.9%
Region				
Europe	5	2.43	1.98–2.98	p = 0.617; I^2^ = 0%
USA	2	2.42	1.95–2.99	p = 0.274; I^2^ = 0%
Gender				
Men	2	2.27	1.63–3.15	p = 0.519; I^2^ = 0%
Men + women	6	2.49	2.11–2.93	p = 0.581; I^2^ = 0%
NT-proBNP value				
Cutoff	4	2.12	1.62–2.77	p = 0.681; I^2^ = 0%
Quintile/Tertile	4	2.60	2.18–3.10	p = 0.716; I^2^ = 0%
Adjustment for renal function				
Yes	5	2.44	2.08–2.85	p = 0.617; I^2^ = 0%
No	3	2.49	1.69–3.69	p = 0.409; I^2^ = 0%
2. Cardiovascular mortality				
Sample size				
≥1,000	4	3.63	2.46–5.37	p = 0.074; I^2^ = 56.8%;
<1,000	4	4.07	2.52–6.56	p = 0.174; I^2^ = 39.7%
Mean age				
≥70 years	2	5.10	3.33–7.81	p = 0.596; I^2^ = 0%
<70 years	6	3.40	2.45–4.70	p = 0.110; I^2^ = 44.3%
Follow-up duration				
>5 years	6	3.39	2.60–4.42	p = 0.174; I^2^ = 35%
≤5 years	2	6.10	3.48–10.50	p = 0.344; I^2^ = 0%
Region				
Europe	5	3.61	2.44–5.36	p = 0.152; I^2^ = 40.3%
USA	2	3.76	2.27–6.25	p = 0.056; I^2^ = 72.6%
Gender				
Men	3	3.02	2.00–4.55	p = 0.230; I^2^ = 32%
Men + women	5	4.56	2.98–6.96	p = 0.064; I^2^ = 54.9%
NT-proBNP value				
Cutoff	2	3.28	1.63–6.60	p = 0.110; I^2^ = 60.8%
Quintile/Tertile	6	4.00	2.83–5.66	p = 0.070; I^2^ = 50.9%
Adjustment for renal function				
Yes	7	3.68	2.69–5.04	p = 0.617; I^2^ = 0%
No	1	4.69	2.53–8.72	—

HR, Hazard ratio; CI, confidence interval; NT-proBNP, N-terminal pro-brain natriuretic peptide.
